# Mutations in Ribosomal Proteins, RPL4 and RACK1, Suppress the Phenotype of a Thermospermine-Deficient Mutant of *Arabidopsis thaliana*

**DOI:** 10.1371/journal.pone.0117309

**Published:** 2015-01-27

**Authors:** Jun-Ichi Kakehi, Eri Kawano, Kaori Yoshimoto, Qingqing Cai, Akihiro Imai, Taku Takahashi

**Affiliations:** Graduate School of Natural Science and Technology, Okayama University, Okayama, Japan; Universidad Miguel Hernández de Elche, SPAIN

## Abstract

Thermospermine acts in negative regulation of xylem differentiation and its deficient mutant of *Arabidopsis thaliana*, *acaulis5* (*acl5*), shows excessive xylem formation and severe dwarfism. Studies of two dominant suppressors of *acl5*, *sac51-d* and *sac52-d*, have revealed that *SAC51* and *SAC52* encode a transcription factor and a ribosomal protein L10 (RPL10), respectively, and these mutations enhance translation of the *SAC51* mRNA, which contains conserved upstream open reading frames in the 5’ leader. Here we report identification of *SAC53* and *SAC56* responsible for additional suppressors of *acl5. sac53-d* is a semi-dominant allele of the gene encoding a receptor for activated C kinase 1 (RACK1) homolog, a component of the 40S ribosomal subunit. *sac56-d* represents a semi-dominant allele of the gene for RPL4. We show that the GUS reporter activity driven by the CaMV 35S promoter plus the *SAC51* 5’ leader is reduced in *acl5* and restored by *sac52-d*, *sac53-d*, and *sac56-d* as well as thermospermine. Furthermore, the *SAC51* mRNA, which may be a target of nonsense-mediated mRNA decay, was found to be stabilized in these ribosomal mutants and by thermospermine. These ribosomal proteins are suggested to act in the control of uORF-mediated translation repression of *SAC51*, which is derepressed by thermospermine.

## Introduction

Polyamines such as putrescine and spermidine are small organic amines present in all living cells. A tetraamine, thermospermine, is a structural isomer of spermine and was first discovered from thermophilic bacteria *Thermus thermophilus* [1]. Thermospermine is distributed widely throughout the plant kingdom [2]. The *acaulis5* (*acl5*) mutant of *Arabidopsis thaliana* shows excessive xylem formation and severe dwarfism [3]. The responsible gene *ACL5* had initially been reported to encode spermine synthase [4] but was later shown to encode thesmospermine synthase [5]. In fact, the *acl5* phenotype is rescued by exogenous treatment with thermospermine but not with spermine [6]. An Arabidopsis mutant defective in a gene encoding spermine synthase, *SPMS*, shows wild-type morphology under normal growth condition [7]. Thus, in vascular plants, thermospermine appears to be specifically required for negative regulation of xylem formation. A previous study of *thickvein* (*tkv*), another mutant allele of *ACL5*, suggests that *ACL5/TKV* is involved in polar auxin transport for proper vein development [8]. Further, a study shows cell-specific expression of *ACL5* in early developing vessel elements and suggests that *ACL5* prevents premature death of developing xylem vessels [9]. A chemical biology approach revealed that the *acl5* phenotype is enhanced by persistent auxin analogs [10]. These results, together with the fact that *ACL5* expression is up-regulated by auxin [4] and down-regulated by thermospermine [6], suggest that thermspermine acts in a negative feedback loop that opposes auxin-induced xylem formation [10]. It was further shown in poplar [11] and Arabidopsis [12] that the class III homeodomain-leucine zipper (HD-ZIP III) transcription factors regulate *ACL5* expression in this feedback context of auxin signaling. Thermospermine, in turn, represses the expression of an auxin response factor (ARF) gene *MONOPTEROS* (*MP*), which is a master regulator for auxin-dependent vascular development, and that of the HD-ZIP III genes [13].

As one approach to study the molecular mechanism of action of thermospermine in plant growth, we have screened for and identified suppressor mutants named *suppressor-of-acl5* (*sac*) from ethyl methanesulfonate (EMS)-mutagenized seeds of *acl5–1* that recover the phenotype without thermospermine [14]. The first mutant *sac51-d* is a dominant allele of the gene for a basic helix-loop-helix (bHLH) transcription factor and completely suppresses the phenotype of *acl5–1*. The 5′ leader sequence of *SAC51* mRNA contains five short upstream open reading frames (uORFs). uORFs often function in regulating the main ORF translation and are present with high frequency in plant mRNAs [15]. Since *sac51-d* has a point mutation that introduces a premature termination codon (PTC) in the 4th uORF of *SAC51*, its inhibitory effect on the main ORF translation is disrupted in *sac51-d* and the dominant trait of *sac51-d* may be attributable to overproduction of the SAC51 protein [14]. This uORF is also conserved in other plant genomes [16]. Such conserved uORFs may be often associated with regulatory genes and have been classified into about 30 groups in angiosperms [17, 18]. In Arabidopsis, for instance, the *bZIP11* mRNA harbors a uORF conserved among *bZIP11* homologs in different plant species. Its encoded small peptide inhibits main ORF translation in response to high sucrose levels probably by stalling the ribosome on the mRNA [19]. Two uORFs of the Arabidopsis *AdoMetDC1* mRNA encoding *S*-adenosylmethionine decarboxylase are involved in polyamine-responsive repression of the main ORF translation [20].

On the other hand, *sac52-d* is a semi-dominant allele of the gene encoding a ribosomal protein L10, RPL10A [21]. RPL10 is a component of the 60S large ribosomal subunit, which is highly conserved in eukaryotes [22], and has been shown in yeast to be a multifunctional translational regulator that operates in the 60S subunit biogenesis, export, and joining with the 40S subunit [23]. *sac52-d* enhances *SAC51* translation in *acl5–1* probably by reducing or eliminating the inhibitory effect of uORFs on the main ORF translation [21]. Given the fact that thermospermine up-regulates the *SAC51* mRNA level [6], *SAC51* may be a key gene that mediates thermospermine signaling. Because most cellular polyamines are bound to RNA [24], it is possible that thermospermine targets *SAC51* mRNA and leads to enhancing its main ORF translation [2]. However, the mode of action of thermospermine in *SAC51* transcription and/or translation remains unknown.

In this study, we show that the genes responsible for two additional suppressor mutants of *acl5–1* designated *sac53-d* and *sac56-d* encode a receptor for activated C kinase 1 (RACK1) homolog and a ribosomal protein L4 (RPL4), respectively. RACK1 is also a component of the 40S ribosomal subunit. Our results show that *SAC51* mRNA may be a target of nonsense-mediated mRNA decay (NMD) in the absence of thermospermine and that these ribosomal mutations enhance translation of the *SAC51* main ORF, thereby stabilizing and accumulating the *SAC51* mRNA. NMD is a eukaryotic mRNA surveillance mechanism that degrades aberrant mRNAs harboring PTC located upstream of the exon-exon boundary, which is marked by the exon junction complex (EJC), and/or a long 3’-UTR [25, 26]. Therefore, uORF-containing mRNAs are likely to be a major class of natural NMD targets. Thermospermine appears to allow the *SAC51* mRNA to bypass the NMD pathway.

## Materials and Methods

### Plant Material and Growth Conditions

Arabidopsis (*Arabidopsis thaliana*) accessions, Columbia (Col-0) and Landsberg *erecta* (L*er*), were used as wild type. *sac52-d* and *sac53-d* were isolated as suppressor mutants of *acl5–1* in the L*er* background [14]. *sac56-d* was identified by an additional screening for *acl5* suppressors from an EMS-mutagenized population of *acl5–1. rack1a-1* and *rack1a-2* were described in [27]. A T-DNA insertion allele of *RPL4A* (Salk_130595), *rpl4a-2* [28], was obtained from the ABRC. *upf1–1* and *upf3–1* were described in Yoine et al [29] and Hori et al [30], respectively.

In most experiments, plants were grown on rock-wool cubes surrounded with vermiculite at 22°C under 16 h light/8 h dark conditions. For RNA preparation, seeds were surface-sterilized with bleach solution containing 0.01% Triton X-100 for 3 min, rinsed three times in sterile water, germinated on MS agar plates supplemented with 3% sucrose, and grown at 22°C under 16 h light/8 h dark conditions.

### Mapping, Cloning, and Genotyping

*sac53-d acl5–1* and *sac56-d acl5–1* in the L*er* background were crossed to *acl5–1* in the Col-0 background. After self-pollination of F1 plants, genomic DNA was extracted from the segregating F2 population and used as template for PCR-based mapping with SSLP and CAPS markers [31, 32]. The Information on these markers was obtained derived from The Arabidopsis Information Resource (TAIR) (http://www.arabidopsis.org). TAIR database was further exploited to identify RFLP markers for fine mapping. The PCR primers designed and used for RFLP were shown in S1 Table. Genes within the chromosomal region delimited by mapping were cloned by PCR from *sac53-d acl5–1* and *sac56-d acl5–1* genomic DNA samples into pGEM-T vector (Promega) to confirm their sequences. DNA sequences were analyzed with an ABI PRISM 310 genetic analyzer (Applied Biosystems).

For generating multiple mutant combinations, genotypes of *acl5–1*, *sac52-d*, *sac53-d*, and *sac56-d* were confirmed by the dCAPS method [33]. Genotypes of T-DNA insertion alleles, *rack1a-1* and *rack1a-2*, were confirmed by PCR using respective gene- and T-DNA-specific primers. Primers and restriction enzymes used are listed in S1 Table.

### Plasmid Construction and Plant Transformation

To test whether or not the suppressor phenotype of *sac56-d* can be recapitulated by transforming *acl5–1* plants with a T-DNA carrying a *sac56-d* genomic DNA fragment, the 2.9-kb DNA fragment containing 893 bp upstream of the start codon, the protein coding region, and 376 bp downstream of the stop codon was amplified from *sac56-d acl5–1* genomic DNA by PCR with primers, 56-F (5’-TTGCT CAGAT TATGG TCCGA-3’) and 56-R (5’-GACAT TTGAA TTCGG TTTGA GCTTC-3’), digested with *Cla*I and *Eco*RI, and cloned in place of the GUS gene in pBI101 (Clontech). For the CaMV 35S promoter-*SAC51* 5’ driving GUS expression, the 5’ leader region of *SAC51* was amplified from wild-type genomic DNA by PCR with primers, 5’-GGATC CGTTT AGACA TTATT GTTCG-3’ and 5’-TCTAG AATCG TCAAA TCGAG TTCC-3’, digested with *Bam*HI and *Xba*I, and inserted between the 35S promoter and the GUS gene of pBI121 (Clontech). Agrobacterium-mediated transformation was performed by the floral-dip method [34].

### GUS Assay

For histochemical staining of GUS activity, samples were prefixed for 20 min in ice-cold 90% (v/v) acetone under vacuum, rinsed three times with water, and incubated in GUS staining buffer (50 mM Na_2_HPO_4_/NaH_2_PO_4_ buffer pH7.0, 2 mM K_3_Fe(CN)_6_, 2 mM K_4_Fe(CN)_6_, 0.1% Triton-X100, 1 mM X-Gluc) at 37°C overnight. Samples were then treated with 70% ethanol to remove chlorophyll. Fluorometric assay of GUS activity was performed as described previously [35]. The fluorescence was measured with an RF-1500 Spectrofluorophotometer (Shimadzu). Total protein content was measured using the Bradford assay (BioRad).

### RNA Extraction and RT-PCR

Plant total RNA was prepared by the SDS-Phenol method [3] and converted to the first strand cDNA using PrimeScript reverse transcriptase (Takara, Kyoto, Japan) and the oligo(dT) primer. Quantitative real-time PCR was performed on the DNA Engine Opticon2 (Bio-Rad) using the Kapa SYBR fast universal qPCR kit (Kapa Biosystems). *UBQ10* was used as an internal control. The relative expression was calculated as ratio between mutants and the wild type or before and after treatment with cordycepin, and then normalized by *UBQ10*. All primers used in this analysis are listed in S1 Table online.

### Polyamine Analysis

Polyamines except for thermospermine were purchased from Nakalai Tesque (Tokyo, Japan). Thermospermine was kindly provided by Dr. Masaru Niitsu. For HPLC, polyamines were extracted from seedlings in 3% perchloric acid and benzoylated according to Naka et al [36]. The resulting samples were injected into a reverse-phase column (TSK-gel ODS-100V, 5 μm, 4.6 × 150 mm, Tosoh, Tokyo, Japan) and eluted with 42% (v/v) acetonitrile at a flow-rate of 0.2 mL/min for 30 min using the Agilent 1120 Compact LC. The benzoyl polyamines were detected at 254 nm.

### Microscopy

To examine vein development in cotyledons, seedlings were cleared with chloral hydrate as described [10] and observed under a light microscope equipped with Nomarski DIC optics (DM5000B, Leica). For tissue sections, samples were fixed, embedded into Technovit 7100 resin (TAAB laboratories), sectioned into 10 μm-thick slices, and stained with toluidine blue.

## Results

### *sac53-d* and *sac56-d* Act in a Semidominant Manner

**Figure 1:**
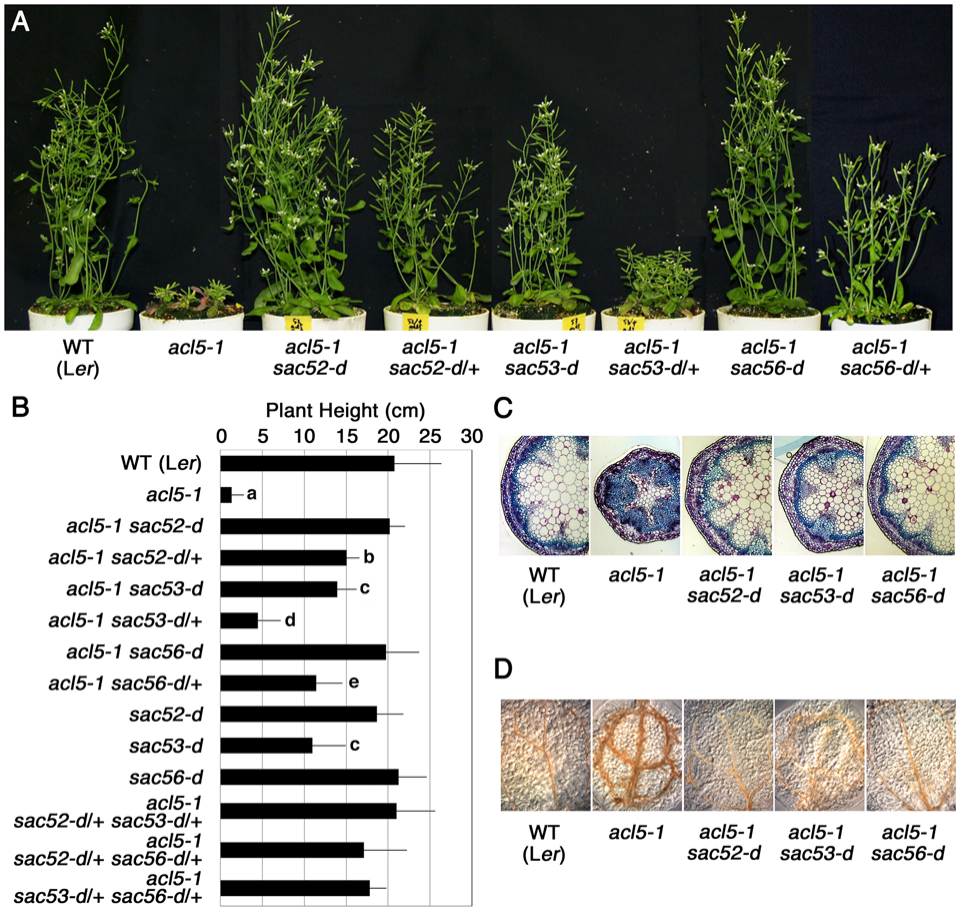
Phenotypes of *acl5–1* and *sac* mutants. **(A)** Gross morphology of 40-day-old plants. **(B)** Plant height of 40-day-old plants. Data show means ± SD (n = 6). Statistical significance was determined by a Student’s t test; significant difference (P < 0.05) from the wild type (L*er*) is indicated by different lowercase letters. **(C)** Stem sections of 40-day-old plants that were stained with toluidine blue. **(D)** Effect of 2,4-D-IOE on cotyledon vein development. Seedlings were grown for 7 days in the liquid MS medium containing 10 μM 2,4D-IOE.

*sac53-d* has previously been isolated as one of *sac* mutants [14]. Mature flowering plants of *acl5–1 sac53-d* recover to about 67% of the wild-type height and those of *acl5–1 sac53-d/+* (*acl5–1* heterozygous for *sac53-d*) show much less recovery (Fig. 1). *sac56-d* was identified in this study by additional screening for *sac* mutants from an EMS-mutagenized population of *acl5–1*. Mature flowering plants of *acl5–1 sac56-d* are almost wild type in appearance and those of *acl5–1 sac56-d/+* recover to about 60% of the wild-type height (Fig. 1A, B). Thus, these alleles appear to act as semidominant traits. The phenotype of *acl5–1 sac56-d* is very similar to that previously observed in *acl5–1 sac52-d* (Fig. 1A) [14]. Transverse sections of stem internodes show that excess xylem differentiation in *acl5–1* is reversed by these suppressor mutations (Fig. 1C). We previously revealed that the isooctyl ester of 2,4-dichlorophenoxyacetic acid (2,4-D IOE) enhances the thick vein phenotype of *acl5–1* [10]. Growth of seedlings in the presence of 2,4-D IOE caused little or no effect on the vein phenotype of *acl5–1 sac52-d* and *acl5–1 sac56-d* cotyledons, and moderately enhanced that of *acl5–1 sac53-d* cotyledons (Fig. 1D). All of *acl5–1 sac52-d*, *acl5–1 sac53-d*, and *acl5–1 sac56-d* were confirmed to contain no detectable thermospermine by HPLC (S1 Fig.).

### *SAC53* Encodes RACK1

**Figure 2:**
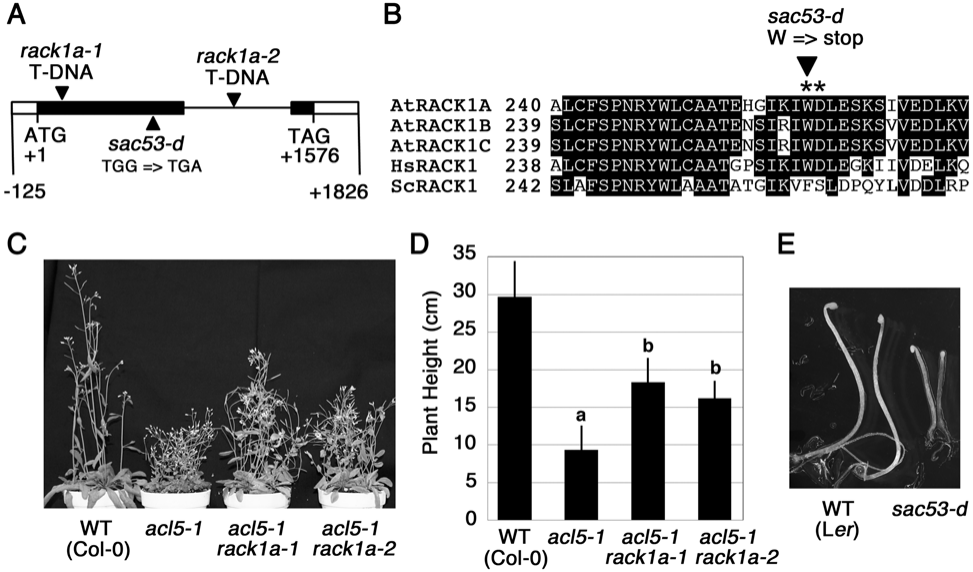
*sac53-d* is an allele of *RACK1A*. **(A)** Exon/intron structure of *RACK1A*. Untranslated and coding regions are shown by white and black boxes, respectively. Arrowheads indicate the position of mutations. **(B)** Comparison of partial amino acid sequences of RACK1. *Arabidopsis thaliana* RACK1A (accession no. NP_173248.1), RACK1B (no. AEE32329), and RACK1C (no. AEE76051) are aligned with *Homo sapiens* RACK1 (no. AAH32006) and *Saccharomyces cerevisiae* RACK1 (no. DAA10013). Shaded boxes indicate conserved amino acids. Asterisks indicate a WD40 domain. **(C)** Gross morphology of 40-day-old plants. **(D)** Plant height of 40-day-old plants. Data show means ± SD (n = 6). Statistical significance was determined by a Student’s t test; significant difference (P < 0.05) from the wild type (Col-0) is indicated by different lowercase letters. **(E)** Gross morphology of 7-day-old etiolated seedlings.

The *SAC53* locus has been mapped on the upper arm of chromosome 1 [14]. Fine mapping experiments delimited the locus to a 100-kb region between markers, T10F20–1 and T10O22–1 (see S1 Table for primer sequences). This 100-kb region contains 32 genes. The nucleotide sequence determination of these genes in *sac53-d acl5–1* revealed a point mutation in At1g18080, one of the three *RACK1* homologous genes in Arabidopsis named *RACK1A* (Fig. 2A). *RACK1A* has one intron and encodes a protein of 327 amino acids containing six WD40 repeats. The G-to-A substitution in *sac53-d* results in a stop codon at the amino acid position 261 within the 6th WD repeat (Fig. 2B).

T-DNA insertion alleles, *rack1a-1* and *rack1a-2*, have been isolated before (Fig. 2A) [27]. To examine whether or not the *acl5–1* phenotype can be also suppressed by *rack1a-1* or *rack1a-2*, we crossed *acl5–1* to these alleles to generate double mutants. Although the *acl5–1* plants heterozygous for *rack1a-1* or *rack1a-2* showed the *acl5* phenotype, both *rack1a-1 acl5–1* and *rack1a-2 acl5–1* partially but apparently recovered the stem growth (Fig. 2C, D). We also confirmed that *sac53-d* single mutants have a slightly reduced height (Fig. 1B) similar to that of *rack1a-1* and *rack1a-2* [27]. In addition, etiolated seedlings of *rack1a-1* and *rack1a-2* have a reduced hypocotyl length [27]. Etiolated seedlings of *sac53-d* also showed a short hypocotyl phenotype (Fig. 2E). Collectively, these results indicate that *sac53-d* represents an allele of *RACK1A*.

### *SAC56* Encodes a Ribosomal Protein L4

**Figure 3:**
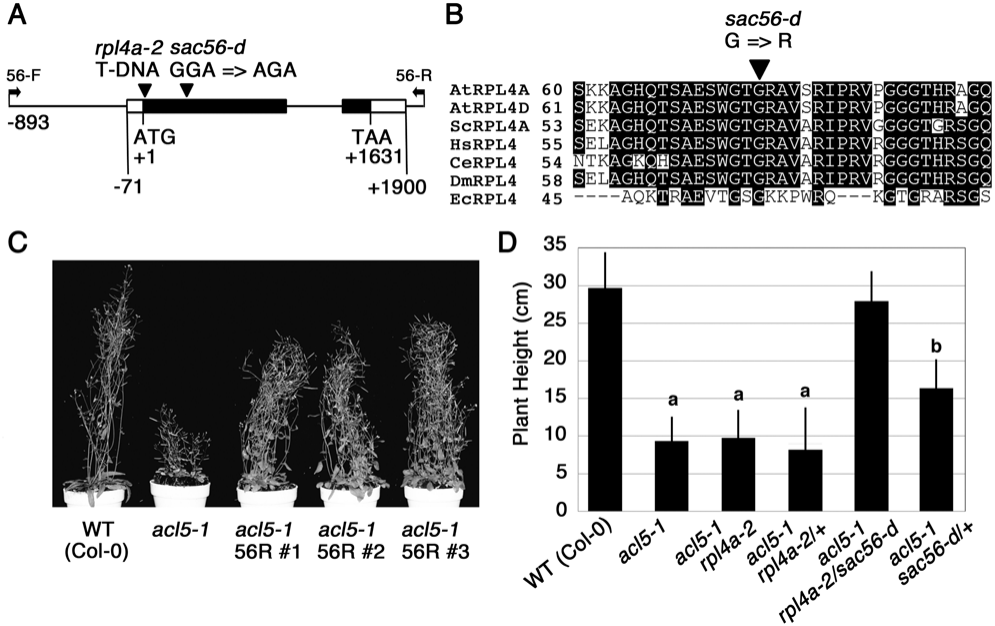
*sac56-d* is an allele of *RPL4A*. **(A)** Exon/intron structure of *RPL4A*. Untranslated and coding regions are shown by white and black boxes, respectively. Arrowheads indicate the position of mutations. Arrows indicate the position of primers used to amplify a *sac56-d* genomic fragment for transgenic recapitulation. **(B)** Comparison of partial amino acid sequences of RPL4. *Arabidopsis thaliana* RPL4A (accession no. AAP37854) and RPL4D (no. AED90529) are aligned with *Oryza sativa* RPL4 (no. NP_001059041), *Saccharomyces cerevisiae* RPL4A (no. NP_009587), *Homo sapiens* RPL4 (no. NP_000959), *Caenorhabditis elegans* RPL4 (no. CCD61249), *Drosophila melanogaster* RPL4 (no. AAG22173), and *Escherichia coli* RPL4 (no. ACI76839). Shaded boxes indicate conserved amino acids. **(C)** Gross morphology of 40-day-old plants. 56R #1 to #3 represent three *acl5–1* lines that were independently transformed with a *sac56-d* genomic fragment shown in **(A)**. **(D)** Plant height of 40-day-old plants. Data show means ± SD (n = 6). *rpl4a-2/sac56-d* indicates a genotype heterozygous for *rpl4a-2* and *sac56-d*. Statistical significance was determined by a Student’s t test; significant difference (P < 0.05) from the wild type (Col-0) is indicated by different lowercase letters.

The *SAC56* locus was mapped on the upper arm of chromosome 3. Fine mapping placed the locus within a ~130-kb interval between markers, F11F8–1 and F8A24–1 (see S1 Table for primer sequences). This region contains 49 genes. Sequencing of these genes in *sac56-d acl5–1* revealed a G-to-A point mutation in At3g09630, which encodes a ribosomal protein L4 named RPL4A (Fig. 3A). The Arabidopsis genome has two active *RPL4* genes, *RPL4A* and *RPL4D*, and two pseudogenes, *RPL4B* and *RPL4C* [37]. *RPL4A* has one intron and encodes a protein of 406 amino acids. The G-to-A base substitution in *sac56-d* changes glycine to arginine at position 75. RPL4 is a highly conserved component of the large ribosomal subunit across kingdoms and contains a globular surface domain and a long ‘tentacle’ that reaches into the core of the large subunit to form part of the lining of the peptide exit tunnel [38]. The glycine residue mutated in *sac56-d* is located within the tentacle and conserved across eukaryotes and prokaryotes (Fig. 3B).

To confirm that the mutation in *RPL4A* is responsible for the suppression of the *acl5–1* phenotype, a genomic DNA fragment containing this gene was amplified by PCR from *sac56-d* and introduced into *acl5–1* by Agrobacterium-mediated transformation. As shown in Fig. 3C and 3D, all three independent transgenic lines showed significant recovery of plant height. We also confirmed that, while a T-DNA insertion allele of *RPL4A*, *rpl4a-2* [28], did not suppress the *acl5–1* phenotype, plants heterozygous for both *sac56-d* and *rpl4a-2* restored the phenotype to the level of wild type (Fig. 3D), suggesting that they are substantially hemizygous for *sac56-d*. It is thus concluded that *sac56-d* represents an allele of *RPL4A*. Unlike *rpl4a-2* which displays narrow pointed first leaves and reduced root elongation [28], the *sac56-d* single mutant was indistinguishable from the wild type in appearance (Fig. 1B and data not shown).

### Phenotypes of Double Mutants

To examine genetic relationships among *sac52-d*, *sac53-d*, and *sac56-d*, we crossed these mutants and generated double trans-heterozygotes in the *acl5–1* background. All of *sac52-d*/+ *sac53-d*/+, *sac52-d*/+ *sac56-d*/+, and *sac53-d*/+ *sac56-d*/+ trans-heterozygotes showed an additive effect with respect to the recovery of plant height in *acl5–1* (Fig. 1B).

**Figure 4:**
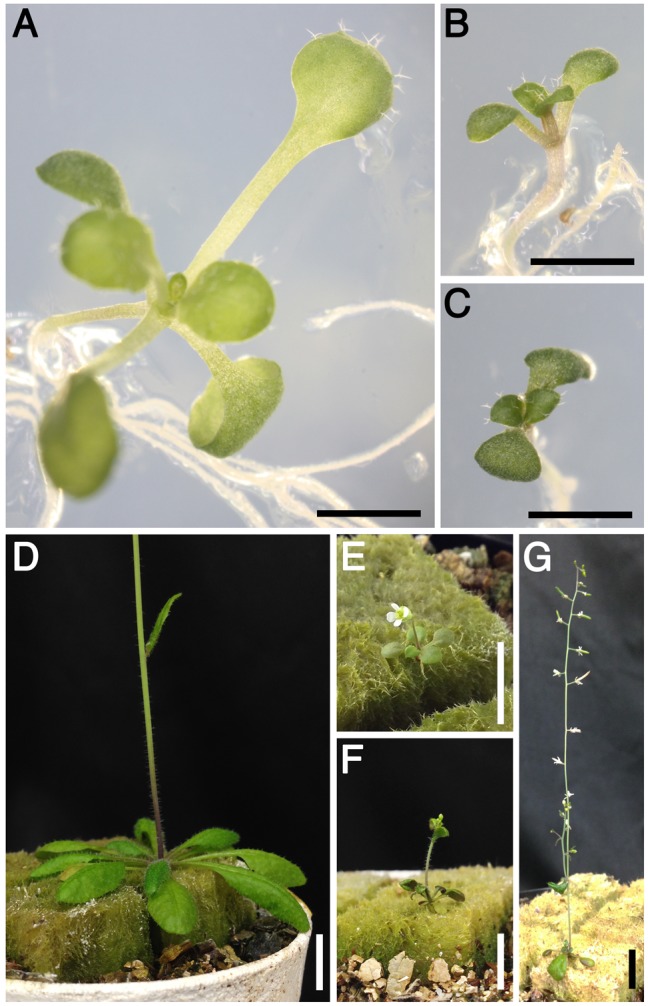
Phenotypes of *sac52-d sac56-d* and *sac53-d sac56-d* mutants. **(A)** A 12-day-old wild-type seedling. **(B)** A 12-day-old *sac52-d sac56-d* seedling. **(C)** A 12-day-old *sac53-d sac56-d* seedling. **(D)** A 30-day-old wild-type plant. **(E)** A 30-day-old *sac52-d sac56-d* plant. **(F)** A 30-day-old *sac53-d sac56-d* plant. **(G)** A 45-day-old *sac53-d sac56-d* plant. Bars = 5 mm in **(A)** to **(C)**, and 10 mm in **(D)** to **(G)**.

We further attempted to make double *sac* homozygous mutants but have so far obtained no plants of *sac52-d sac53-d*. Because the distance between *SAC52/RPL10A* and *SAC53/RACK1A* is approximately 1330 kb on chromosome 1, they may be too close to each other, otherwise *sac52-d sac53-d* double mutations might be lethal to gametophytes. On the other hand, *sac52-d sac56-d* seedlings were very small and often displayed growth arrest before bolting (Fig. 4A, B). *sac53-d sac56-d* seedlings also showed a phenotype of extremely small size similar to that of *sac52-d sac56-d* (Fig. 4C). *sac52-d sac56-d* rarely formed flowers while *sac53-d sac56-d* formed flowers more frequently and produced only a few seeds (Fig. 4D-G).

### Effect of *sac* Mutations on Gene Expression

**Figure 5:**
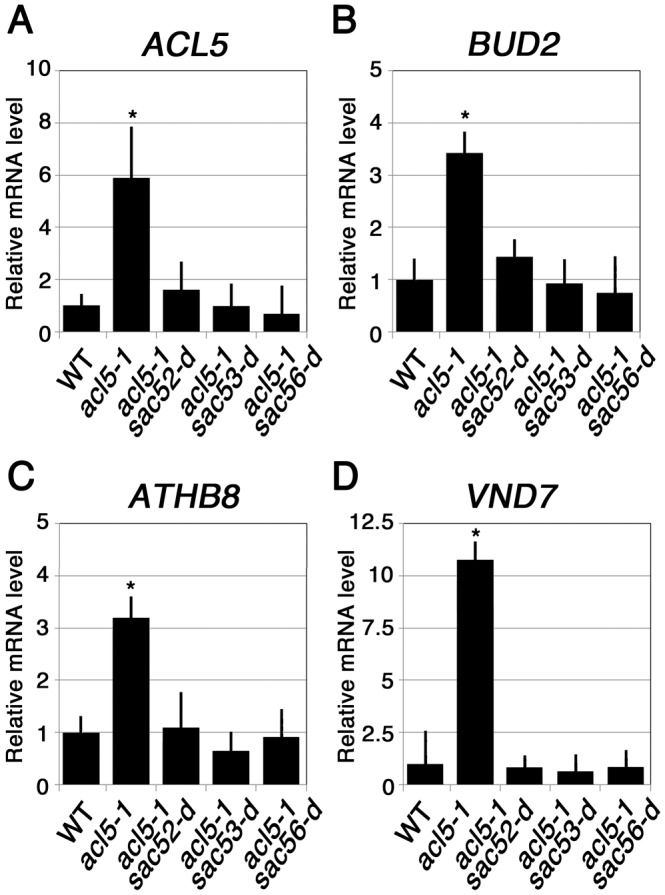
Effect of *sac* mutations on gene expression. **(A)** to **(D)** Relative mRNA levels of *ACL5* **(A)**, *BUD2/AdoMetDC4* **(B)**, *ATHB8* **(C)**, and *VND7* **(D)** were examined by quantitative RT-PCR. Seedlings were grown for 10 days in MS agar plates. mRNA levels were normalized to the *UBQ10* mRNA level and set to 1 in wild type (WT). Data show means ± SD (n = 3). Asterisks indicate significant differences (P < 0.05) from the wild type (Col-0).

We examined the effect of these ribosomal mutations on the expression of genes related to thermospermine biosynthesis, *ACL5* and *BUD2/AdoMetDC4*, and the regulation of xylem differentiation, *ATHB8* and *VND7. BUD2/AdoMetDC4* encodes an S-adenosylmethionine decarboxylase, which may act specifically in the synthesis of thermospermine to provide the aminopropyl donor [13]. *ATHB8* is a member of the HD-ZIP III family that plays a key role in xylem differentiation [39] and has recently been shown to directly regulate *ACL5* expression [12]. *VND7* is a NAC-family transcription factor gene that controls xylem vessel element differentiation [40]. Previous studies have shown that expression of all these genes were up-regulated in *acl5–1* and down-regulated by exogenous treatment with thermospermine [6, 13]. Our RT-PCR experiments revealed that mRNA levels of these genes in *acl5–1* were reduced to normal level by *sac52-d*, *sac53-d*, and *sac56-d* (Fig. 5).

### *SAC51* 5’-*GUS* Expression in *sac* Mutants

**Figure 6:**
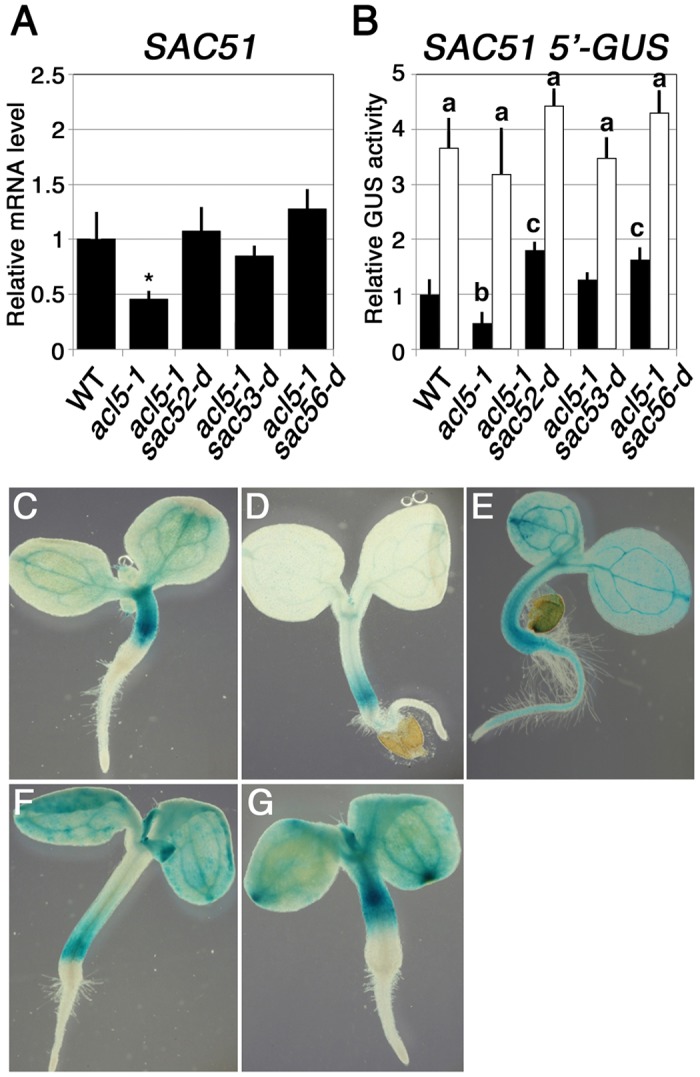
Effect of *sac* mutations on the *SAC51* 5’-*GUS* expression. **(A)** Effect of *sac* mutations on *SAC51* expression. Relative mRNA levels of *SAC51* in 10-day-old seedlings were examined as shown in the Fig. 5 caption. An asterisk indicates a significant difference (P < 0.05) from the wild type (L*er*). **(B)** Effect of thermospermine on CaMV 35S promoter-driven *SAC51* 5’-*GUS* expression. Seedlings carrying the *SAC51* 5’ leader-*GUS* gene fused with the CaMV 35S promoter were grown for 10 days in MS agar plates and incubated for 24 h in MS solutions without (black bars) or with 100 μM thermospermine (white bars). Data show means ± SD (n = 3). Significant difference (P < 0.05) from the wild type (L*er*) is indicated by different lowercase letters. **(C)** to **(G)** GUS staining of wild-type **(C)**, *acl5–1* **(D)**, *acl5–1 sac52-d* **(E)**, *acl5–1 sac53-d* **(F)**, and *acl5–1 sac56-d* **(G)** seedlings carrying the *SAC51* 5’ leader-*GUS* gene fused with the CaMV 35S promoter. Seedlings were grown for 3 days in MS agar plates.

We further examined *SAC51* expression in each *sac* mutant and the result showed that the *SAC51* mRNA level, which is reduced in *acl5–1*, is recovered by *sac52-d*, *sac53-d*, and *sac56-d* (Fig. 6A). *sac52-d* enhances the *SAC51* main ORF translation [21]. To examine whether *sac53-d* and *sac56-d* also affect the *SAC51* translation or not, we generated transgenic lines carrying the GUS reporter gene under the control of the CaMV 35S promoter plus the *SAC51* 5’ leader sequence and the construct was introduced into each mutant by crosses. While the 35S promoter is not responsive to themospermine (S2 Fig.), the GUS activity derived from the 35S-*SAC51* 5’-GUS construct was about two-fold lower in *acl5–1* seedlings than in wild-type seedlings and was increased to similar levels in both *acl5–1* and wild type after one-day treatment with thermospermine (Fig. 6B), indicating that the *SAC51* 5’ leader sequence is responsive to thermospermine. The GUS activity in *sac52-d*, *sac53-d*, and *sac56-d* in the *acl5–1* background was higher than that in the wild type and further showed an increase by one-day treatment with thermospermine (Fig. 6B). Observation of the GUS staining pattern revealed that most above-ground tissue of the seedling was stained in these mutants but only veins were preferentially stained in the wild type without thermospermine treatment whereas only faint staining was detected in *acl5–1* (Fig. 6C-G).

### *SAC51* mRNA Is Stabilized by Thermospermine and *sac* Mutations

**Figure 7:**
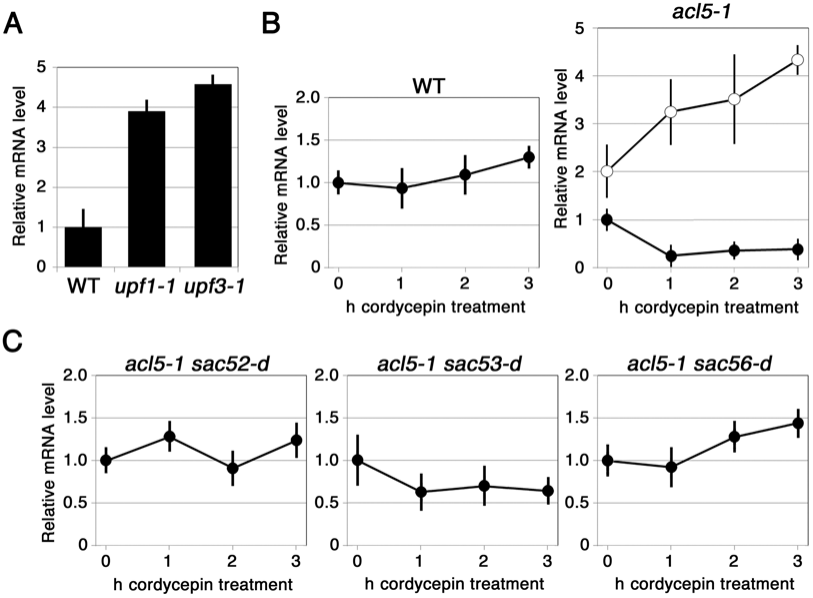
Effect of thermospermine and *sac* mutations on the *SAC51* mRNA stability. **(A)** Effect of *upf1–1* and *upf3–1* on *SAC51* expression. **(B)** Time-course assays of *SAC51* mRNA stability in wild type and *acl5–1*. Seedlings were treated with 0.6 mM cordycepin for indicated periods of time. In *acl5–1*, closed circles and open circles indicate data on seedlings treated with cordycepin and those pre-treated with 100 μM thermospermine for 1 h before addition of cordycepin, respectively. **(C)** Time-course assays of *SAC51* mRNA stability in *acl5–1 sac* double mutants. In **(A)** to **(C)**, seedlings were grown for 10 days in MS agar plates. Relative mRNA levels of *SAC51* were examined as shown in the Fig. 5 caption and set to 1 in wild type **(A)** and in each seedling before treatment (**[B]** and **[C]**).

The Arabidopsis genome contains three additional genes with high sequence homology to *SAC51*, named *SACL1* (At5g09460), *SACL2* (At5g50010), and *SACL3* (At1g29950) [14]. These mRNAs have been shown to accumulate to higher levels in the *low-beta-amylase1* (*lba1*) mutant than in the wild type [29]. The gene responsible for *lba1* encodes an RNA helicase UPF1 involved in nonsense-mediated mRNA decay (NMD). Another study has also suggested that *SAC51* and its homologs are NMD target genes because of the presence of conserved uORFs within the 5’ leader of these mRNAs [41]. We confirmed that the *SAC51* mRNA level was approximately four-fold higher in *lba1* (*upf1–1*) than in the wild type (Fig. 7A). The *SAC51* mRNA level was also higher in *upf3–1*, a mutant for *UPF3* which is another factor required for NMD [30], than in the wild type (Fig. 7A). Then we performed time-course assays of mRNA stability by using cordycepin, a nucleoside analog that inhibits transcription elongation, and the results revealed that the *SAC51* mRNA was destabilized in *acl5–1* and the stability was markedly restored by pretreatment with thermospermine (Fig. 7B). On the other hand, the *SAC51* mRNA level was not affected in *sac52-d acl5–1* and *sac56-d acl5–1* after cordycepin treatment while it was moderately declined in *sac53-d acl5–1* (Fig. 7C).

## Discussion

*SAC51* consists of four exons and three introns that interrupt an 870-base long 5’ leader region. The 4th exon contains a complete coding sequence of a bHLH protein followed by an approximately 550-base long 3’ untranslated region. The 5’ leader region contains five uORFs encoding 20, 16, 48, 53, and 6 amino acid peptides, respectively, among which the 1st and 4th ones are highly conserved between different plant species [15]. As shown in a previous study [41], the *SAC51* mRNA level is increased in two mutants defective in NMD, *upf1* and *upf3*. UPF1, UPF2, and UPF3 are all essential for NMD [42]. In plants, a 13 amino acid long uORF of the Arabidopsis *AtMHX* mRNA, which encodes a vacuolar metal transporter, has been shown to trigger NMD [43]. Although even a 31 amino acid long uORF can fail to activate the NMD response, plant uORFs may generally induce NMD in a size-dependent manner [44]. Taking together with the fact that the *SAC51* 4th uORF appears to be inhibitory to the main ORF translation and the level of the *sac51-d* mRNA in *sac51-d* is much higher than that of the *SAC51* mRNA in wild type [14], the NMD response of *SAC51* may be triggered by the ribosome that translates *SAC51* 4th uORF and leads the NMD machinery to recognize its downstream EJC. In *sac51-d*, the mutated 4th uORF of three amino acids would be too short to trigger NMD. Our experiments using cordycepin as a transcriptional inhibitor revealed that thermospermine stabilizes the *SAC51* mRNA. We further confirmed by using the 35S promoter-*SAC51* 5’ leader-*GUS* gene construct that the *SAC51* 5’ leader region is responsive to thermospermine. It is therefore likely that the uORF-mediated NMD of *SAC51* mRNA occurs in *acl5–1* and in wild type tissues containing no thermospermine. We have also generated *acl5–1 upf1* and *acl5–1 upf3* but these *upf* mutations did not suppress the *acl5* phenotype (data not shown). It might be difficult for normal ribosomes to reinitiate translation of the *SAC51* main ORF in the absence of thermospermine. Alternatively, there might be an additional mechanism that represses the *SAC51* translation.

In addition to previously identified *sac52-d*, representing a semi-dominant allele of *RPL10A* [21], *sac53-d* and *sac56-d* are also semi-dominant alleles of ribosomal protein genes, *RACK1A* and *RPL4A*, respectively. Our results indicate that all of these ribosomal mutations suppress thermospermine deficiency of *acl5* by positively affecting translation of the *SAC51* main ORF and its mRNA stability. Semi-dominant nature of these alleles might be explained by a positive effect of the ribosome containing one of these defective components on the main ORF translation. Although there are some exceptions, e.g. in yeast, NMD occurs in an initial “pioneer” round of translation of PTC-containing mRNAs that retain downstream EJCs but not in subsequent rounds because EJCs are removed in the pioneer round by a scanning ribosome so as to preclude NMD of qualified mRNAs [45]. Thus, once a *SAC51* mRNA is translated in the pioneer round by a mutant ribosome in heterozygous *sac52-d*, *sac53-d*, or *sac56-d*, the mRNA presumably becomes immune to NMD and enables translation reinitiation from the main AUG in subsequent rounds.

While these suppressors have a common positive effect on *SAC51* translation, both *sac52-d sac56-d* and *sac53-d sac56-d* double mutants show severe growth defects (Fig. 4), suggesting functional interactions between these responsible proteins. RACK1 is a beta-propeller scaffold protein comprising seven WD40 repeats, which was originally identified as an anchoring protein for protein kinase C and has been implicated in mediating various signal transduction pathways by interacting with a number of signaling molecules [46, 47]. But rather RACK1 is known to be a core component of the 40S ribosomal subunit located near the mRNA exit channel and contact with 18S rRNA [48]. RACK1 is also involved in nascent peptide-dependent ribosome stalling to trigger the no-go-mediated mRNA decay (NGD) response [49], which is another eukaryotic mRNA quality control mechanism additional to NMD [50]. NGD may occur in plants in a similar way to that in mammalian and yeast cells because such factors as Dom34 and Hbs1, which function in initial recognition of stalled ribosomes in the NGD system, are conserved in plants [51]. Although in contrast to the well-known effect of polyamines on ribosome stalling at the uORF of *AdoMetDC* translation [52], it is conceivable that the nascent peptide by the *SAC51* 4th uORF causes ribosome stalling in the absence of thermospermine and triggers NGD but does not in *sac53-d*. The result that *sac53-d* had a relatively weak effect on the *SAC51* mRNA stability compared to *sac52-d* and *sac56-d* might reflect the difference between the process involving RACK1 and that involving RPL10 and RPL4. Additional work will be required to determine whether or not specific mRNAs can be subjected to both NMD and NGD in plants.

The *Arabidopsis* genome has three homologs of *RACK1* designated *RACK1A*, *B*, and *C*. These three RACK1 isoforms have been shown to physically interact with eukaryotic initiation factor 6 (eIF6), a key regulator of 80S ribosome assembly [53]. Two T-DNA insertion mutants of *RACK1A* used in this study, *rack1a-1* and *rack1a-2*, display pleiotropic phenotypes including growth defects and altered responses to plant hormones [27]. Because these phenotypes are more serious than those of *rack1b* and *rack1c, RACK1A* may represent a major member of the family [54]. Given that uORFs are present in over 30% of Arabidopsis mRNAs [15], the phenotypes might also be due in part to increase in translation of mRNAs including *SAC51* mRNA that is normally under tight control by uORFs. It remains to be examined whether or not *sac53-d* affects translation of uORF-containing mRNAs in general.

The third ribosomal component that affects *SAC51* mRNA translation was found to be RPL4. RPL4 is a highly conserved constituent of the large ribosomal subunit. An analysis of the crystal structure of the bacterial ribosome suggests that RPL4 and RPL22 (RPL17 in eukaryotes) are located near the constricted region of the nascent peptide exit tunnel of the ribosome [38]. RPL4 and RPL22 contain elongated “tentacles” that reach into the peptide exit tunnel. The translational arrest caused by ribosomal stalling in a number of regulatory peptide sequences is released by mutations of these domains of RPL4 and RPL22 [55]. The fungal arginine attenuator peptide, which is encoded by a uORF of *Arg-2* and causes ribosome stalling in response to arginine, has been shown to interact with RPL4 and RPL17 during translation in the ribosome [56]. According to the effect of *sac56-d* and *sac56-d/rpl4a-2* mutations on the *acl5* phenotype, *sac56-d* may represent a gain-of-function allele. It is possible that the amino acid alteration within the tentacle domain of RPL4 in *sac56-d* affects conformation of the exit tunnel to preclude ribosome stalling.

Loss-of-function mutants of *RPL4A*, which were initially identified as a mutant with altered trafficking of vacuolar targeted proteins, display morphological phenotypes such as narrow pointed first leaves, an abnormal cotyledon number, short roots, and short hypocotyls, suggesting the effect of a failure in the auxin-mediated ribosome biogenesis [28]. The *rpl4d* mutant was also identified as having pointed leaves [57]. Genetic crosses of these mutants indicate that the mutant phenotypes are dose-sensitive and that two active copies of *RPL4*, independent of their identities, are essential for plant viability [28]. T-DNA insertion mutations of *RPL4D* and also of *RPL5A* result in a decreased translation of the ARF genes, most of which contain uORFs [58]. Taken together with our result of *sac56-d*, RPL4 appears to be involved in translation of both the uORF and the main ORF.

Emerging evidence suggests that mutations in ribosomal protein genes affect specific aspects of plant development [59], suggesting that each ribosomal protein has a unique role associated with different developmental processes. Studies of *short valve1* (*stv1*), a mutant of *RPL24B*, have revealed that RPL24B is required for translation reinitiation of the main ORF of the ARF genes *ETTIN* and *MP*, both of which contain uORFs [60–62]. *RPL10B* and *RPL10C* have been shown to be involved in translation under ultraviolet-B stress [63, 64]. Here our results revealed that RPL4 and RACK1 as well as RPL10 have a role in the control of uORF-mediated translation repression of the *SAC51* mRNA, which is derepressed by thermospermine. However, the process how ribosomes reach the main AUG and reinitiate translation is far from clear. Further study of additional *sac* mutations will help to unravel the mechanism of thermospermine-dependent translation.

### Accession Numbers

Sequence data from this article can be found in the Arabidopsis Genome Initiative database under the following accession numbers: *ACL5* (At5g19530); *SAC51* (At5g64340); *SAC52/RPL10A* (At1g14320); *SAC53/RACK1A* (At1g18080); *RACK1B* (At1g48630); *RACK1C* (At3g18130); *SAC56/RPL4A* (At3g09630); *RPL4D* (At5g02870); *UPF1* (At5g47010); *UPF3* (At1g33980); *BUD2/AdoMetDC4* (At5g18930); *ATHB8* (At4g32880); *VND7* (At1g71930);*UBQ10* (At4g05320).

## Supporting Information

S1 Fig HPLC analysis of polyamines extracted from each mutant seedling. Plants were grown for 10 days in MS agar plates. Polyamines were extracted and benzoylated as described [36]. Arrows and asterisks indicate positions of thermospermine and spermine, respectively.
(JPG)

S2 Fig Effect of thermospermine on CaMV 35S promoter-driven *SAC51* 5’-*GUS* expression. Seedlings carrying the *GUS* gene fused with the 35S promoter plus *SAC51* 5’ leader or the solo 35S promoter were grown for 10 days in MS agar plates and incubated for 24 h in MS solutions without (black bars) or with 100 μM thermospermine (white bars). Data show means ± SD (n = 3).
(JPG)

S1 Table List of primers used for RT-PCR, genotyping, and mapping. (DOCX)
