# Supplementary figures and images for: Mutations in Ribosomal Proteins, RPL4 and RACK1, Suppress the Phenotype of a Thermospermine-Deficient Mutant of Arabidopsis thaliana
Source: PLoS One. 2015 Jan 27;10(1):e0117309. doi: 10.1371/journal.pone.0117309 (PMC4308196; doi:10.1371/journal.pone.0117309)

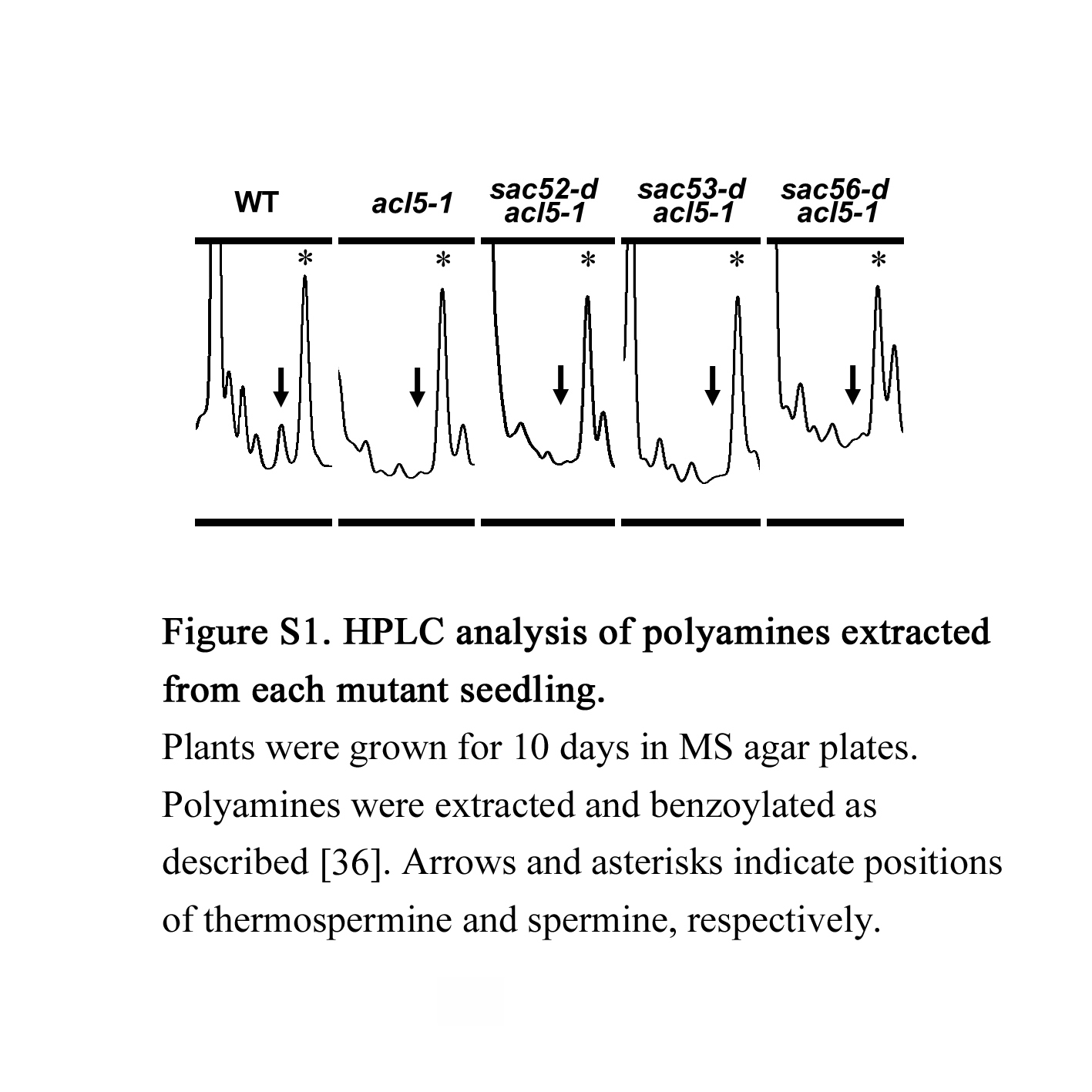

Supplement: S1 Fig — Plants were grown for 10 days in MS agar plates. Polyamines were extracted and benzoylated as described [36]. Arrows and asterisks indicate positions of thermospermine and spermine, respectively. (JPG) [file pone.0117309.s001.jpg]

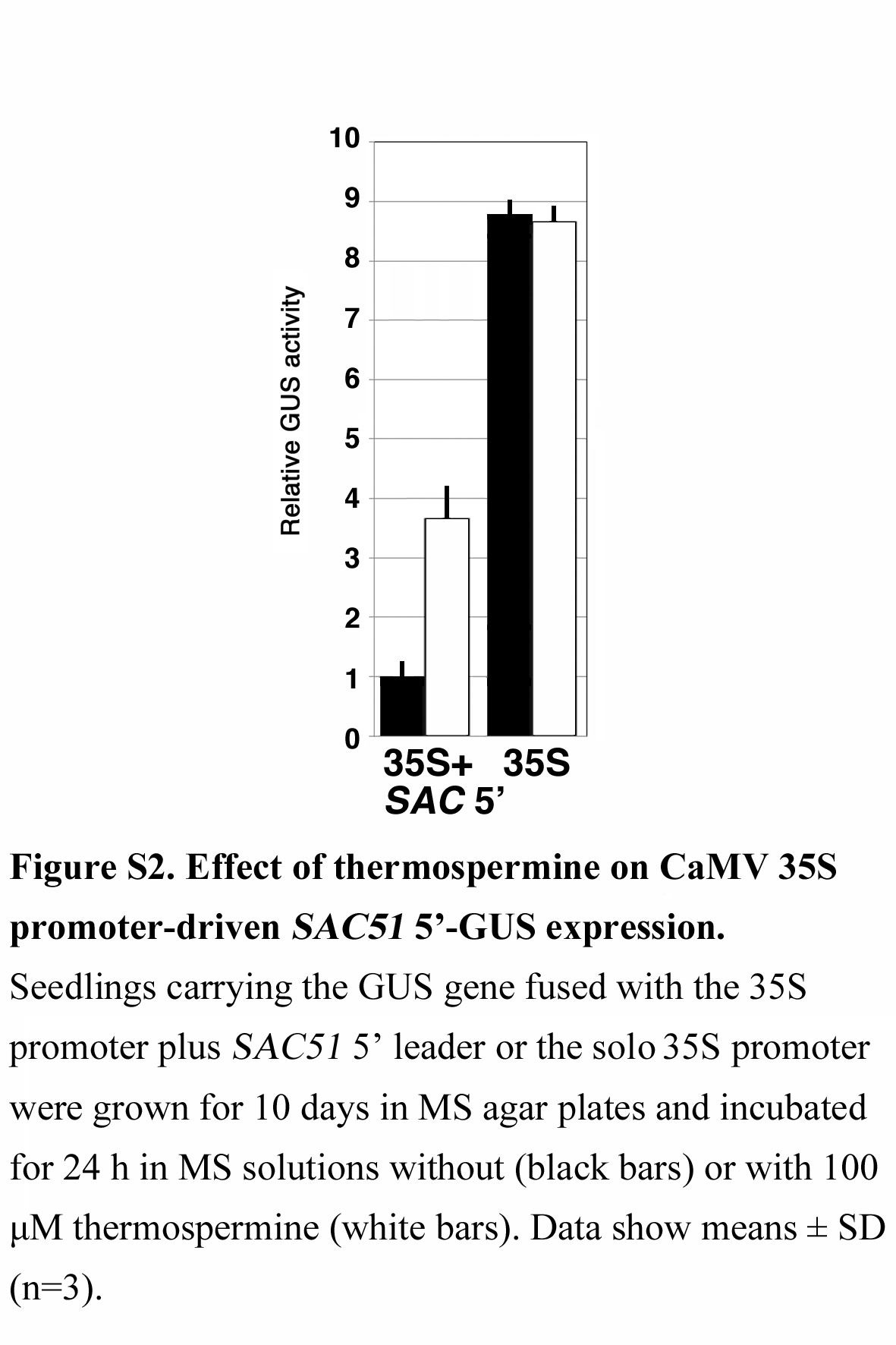

Supplement: S2 Fig — Seedlings carrying the GUS gene fused with the 35S promoter plus SAC51 5’ leader or the solo 35S promoter were grown for 10 days in MS agar plates and incubated for 24 h in MS solutions without (black bars) or with 100 μM thermospermine (white bars). Data show means ± SD (n = 3). (JPG) [file pone.0117309.s002.jpg]
