# Supplementary material for: Mutations in Ribosomal Proteins, RPL4 and RACK1, Suppress the Phenotype of a Thermospermine-Deficient Mutant of Arabidopsis thaliana
Source: PLoS One. 2015 Jan 27;10(1):e0117309. doi: 10.1371/journal.pone.0117309 (PMC4308196; doi:10.1371/journal.pone.0117309)
Supplement: S1 Table — (DOCX) [file pone.0117309.s003.docx]

| Gene or marker name | | DNA sequence | Purpose | Restriction enzyme | Fragment length (bp) |
| --- | --- | --- | --- | --- | --- |
| UBQ10 | F | 5’-CACACTCCACTTGGTCTTGCGT-3’ | RT-PCR |  |  |
|  | R | 5’-AAGATCAACCTCTGCTGGTCCG-3’ |  |  |  |
| ACL5 | F | 5'-ACCGTTAACAGCGATGCTTT-3' |  |  |  |
|  | R | 5'-CCGTTAACTCTCTCTTTGATTC-3' |  |  |  |
| SAC51 | F | 5'-AATTGCCAGGCTGAGTACTT-3' |  |  |  |
|  | R | 5'-GACCGACCTACTATATCCTT-3' |  |  |  |
| BUD2 | F | 5'-ATGGCAGTGTCTGGGTTCGA-3' |  |  |  |
|  | R | 5'-CTATTTCCGACGAGGCGTGA-3' |  |  |  |
| ATHB8 | F | 5'-AGCGTTTCAGCTAGCTTTTGAG-3' |  |  |  |
|  | R | 5'-CAGTTGAGGAACATGAAGCAGA-3' |  |  |  |
| VND7 | F | 5'-CGATGCATCAATATGGCAAC-3' |  |  |  |
|  | R | 5'-AGGGAAGCATCCAAGAGAAT-3' |  |  |  |
| ACL5  -dCAPS | F | 5'-GGAGGTGAAGGCTCTGCTGCTCGA-3' | Genotyping | *Xho*I | WT: 215, 25  *acl5-1*: 240 |
|  | R | 5'-TTTGTTACAGAAAGCATCGCTGTTAAC-3' |  |  |  |
| SAC52  -dCAPS | F | 5’-TGCGAGGTGTTACCGTCAGATCTAG-3’ |  | *Xba*I | WT: 160  *sac52-d*: 125, 25 |
|  | R | 5’-CCATGACACCAAATGGACACAG-3 |  |  |  |
| SAC53  -dCAPS | F | 5’-GAGCACTGTGGCTGTATCACC-3’ |  | *Bgl*II | WT: 220  *sac53-d*: 195, 25 |
|  | R | 5’-CTCAACAATGCTCTTGCTCTCAAGATC-3’ |  |  |  |
| SAC56  -dCAPS | F | 5'-GAGACGGAGAGTGAGAGAAG-3' |  | *Ava*II | WT: 275, 25  *sac56-d*: 300 |
|  | R | 5'-GAGGGATACGAGACACGGCAGGTC-3' |  |  |  |
| RACK1A | F- | 5'-GGCATCTCCAGACACCGAAA-3' |  |  | WT: 1,700  *rack1a-1*: 1,500 (R+pBI-LB)  *rack1a-2*: 500 (R+pBI-LB) |
|  | R- | 5'-GCAGAGAGCAACGACAGC-3' |  |  |  |
| pBI-LB | | 5'-AACCAGCGTGGACCGCTTGCTG-3' |  |  |  |
| RPL4A | F | 5’-GAGACGGAGAGTGAGAGAAG-3’ |  |  | WT: 550  *rpl4a-2*: 500 (R+pBI-LB) |
|  | R | 5’-ACTCACAACAAGAGGCATCTCA-3’ |  |  |  |
| T10F20-1 | F | 5’-TCCGAGACATCATTGGGGTA-3’ | Mapping of *sac53-d* | *Eco*RV | Col-0: 205  Ler: 180, 25 |
|  | R | 5’-GTCTCTGTTCCATAATCCAGGGA-3’ |  |  |  |
| T10O22-1 | F | 5’-TTGCTGGGTGACTTGGAT-3’ |  | *Bss*HII | Col-0: 225  Ler: 200, 25 |
|  | R | 5’-GCTCTGGTTTCGCTCTTT-3’ |  |  |  |
| F11F8-1 | F | 5'-TGTTTGAGACTCGTCACAAG-3' | Mapping of *sac56-d* | *Msp*I | Col-0: 350, 270  Ler: 620 |
|  | R | 5'-ATACCTGGTTCCAGTTCCTT-3' |  |  |  |
| F8A24-1 | F | 5'-GTCACGGATAATCTTCTTGG-3' |  | *Bsp*HI | Col-0: 600  Ler: 440, 160 |
|  | R | 5'-GACCAATATGGTATGGGTTC-3' |  |  |  |

**Table S1. List of primers used for RT-PCR, genotyping, and mapping.**
